# From Parasitized to Healthy-Looking Ants (Hymenoptera: Formicidae): Morphological Reconstruction Using Algorithmic Processing

**DOI:** 10.3390/life12050625

**Published:** 2022-04-22

**Authors:** Sándor Csősz, Ferenc Báthori, Mathieu Molet, Gábor Majoros, Zoltán Rádai

**Affiliations:** 1 Evolutionary Ecology Research Group, Institute of Ecology and Botany, Centre for Ecological Research, 2163 Vácrátót, Hungary; bathori.ferenc@ecolres.hu; 2 MTA-ELTE-MTM Ecology Research Group, Eötvös Loránd University, 1053 Budapest, Hungary; 3 Institute of Ecology and Environmental Sciences-Paris (iEES-Paris), Sorbonne Université, Université Paris Est Créteil, Université Paris Diderot, CNRS, INRAE, IRD, F-75005 Paris, France; mathieu.molet@upmc.fr; 4 Department of Parasitology and Zoology, Faculty of Veterinary Sciences, Szent István University, István u. 2., 1078 Budapest, Hungary; majoros.gabor@univet.hu; 5 Lendület Seed Ecology Research Group, Institute of Ecology and Botany, Centre for Ecological Research, 2163 Vácrátót, Hungary; radai.zoltan@ecolres.hu

**Keywords:** cysticercoid, morphometry, *Temnothorax*, parasitogenic phenotypes, machine learning

## Abstract

Background: Parasites cause predictable alternative phenotypes of host individuals. Investigating these parasitogenic phenotypes may be essential in cases where parasitism is common or taxa is described based on a parasitized individual. Ignoring them could lead to erroneous conclusions in biodiversity-focused research, taxonomy, evolution, and ecology. However, to date, integrating alternative phenotypes into a set of wild-type individuals in morphometric analysis poses extraordinary challenges to experts. This paper presents an approach for reconstructing the putative healthy morphology of parasitized ants using algorithmic processing. Our concept enables the integration of alternative parasitogenic phenotypes in morphometric analyses. Methods: We tested the applicability of our strategy in a large pool of Cestoda-infected and healthy individuals of three *Temnothorax* ant species (*T. nylanderi*, *T. sordidulus*, and *T. unifasciatus*). We assessed the stability and convergence of morphological changes caused by parasitism across species. We used an artificial neural network-based multiclass classifier model to predict species based on morphological trait values and the presence of parasite infection. Results: Infection causes predictable morphological changes in each species, although these changes proved to be species-specific. Therefore, integrating alternative parasitogenic phenotypes in morphometric analyses can be achieved at the species level, and a prior species hypothesis is required. Conclusion: Despite the above limitation, the concept is appropriate. Beyond parasitogenic phenotypes, our approach can also integrate morphometric data of an array of alternative phenotypes (subcastes in social insects, alternative morphs in polyphenic species, and alternative sexes in sexually dimorphic species) whose integrability had not been resolved before.

## 1. Introduction

A large proportion of the 1.9 million extant described species is known only from a single report. These can either be singletons, i.e., species recognized from a single specimen, or uniques, i.e., species represented by multiple specimens but collected once only in history [1]. For example, 30% of all arthropod species are represented by a single specimen [1]. These “oncers” might reflect true rarity [2,3] either because of a limited geographical distribution [3,4], or a low sampling effort, e.g., if they live in exotic or barely accessible habitats (e.g., [5,6]). These might also be alternative phenotypes of a known species developed in response to various environmental factors [7,8], including parasitism [9]. Some parasites of ants and many other insects are known to alter morphological traits of their hosts to various extents, from limited morphological shifts in a single trait to complete *shape-shifts* that alter many traits simultaneously [10]. Polyphenism (i.e., the ability to develop multiple discrete phenotypes from a single genotype) is the hallmark of every ant species. Ants develop very different phenotypes in a single colony, called castes and subcastes, through fine-tuned developmental processes. This feature makes them vulnerable to developing scrambled trait combinations [11] when facing strong environmental disturbances such as parasite infections [12]. These morphological shifts make the infected “parasitogenic” ants look strikingly and consistently different from the typical phenotype of their species [13,14], misleading taxonomists into describing them as new species (e.g., [15,16]).

Beyond the problematic inflation in the number of described species, parasitogenic phenotypes interfere with the accuracy of our research across many key fields in biology, including conservation biology, organismal biology, developmental biology, ecology, evolution, and systematics. To date, omission from data analyses has been the usual practice for handling parasitogenic specimens in research. However, in biodiversity studies, taxonomy, and systematics, particularly in cases where the taxon was named and described after a parasitogenic specimen, these individuals cannot be overlooked. Therefore, it is paramount to associate parasitogenic specimens with their appropriate species using genetics or morphometry.

In this paper, we present a method to re-integrate parasitogenic individuals in the bulk of wild-type individuals via transformation of morphometric data using a model-based algorithmic approach. Our concept relies on the idea that parasites may consistently affect the morphological traits of their host (same extent and direction), resulting in a within-species phenotypic variability of infected individuals that is similar to that of uninfected individuals [17]. In that case, analyzing the morphology of both infected and uninfected specimens can be a helpful tool [12,18]. Our concept would, of course, be harder to apply to species where parasites would randomly affect the morphological traits of their host one way or the other, thus, increasing variance in the overall host population [19].

We selected three *Temnothorax* ant species [*T. nylanderi* (Foerster, 1850), *T. sordidulus* (Müller, 1923), and *T. unifasciatus* (Latreille, 1798)] with a statistically sound number of uninfected workers and Cestoda-infected nestmates. We evaluated the within-species and between-species convergence of the shape-shift caused by infection. In addition, we assessed the frequency distribution of both infected and uninfected individuals. The *Temnothorax*-Cestoda system is undoubtedly an excellent testing ground for our purpose because (i) this infection is well-known for myrmecologists [10,13,20,21,22], (ii) the infected ants are easy to identify in the field; the infection causes faded coloration in *Temnothorax* ants, turning individuals from a typical brown color to a yellowish one [23], and (iii) these parasites are expected to cause similar alternative morphologies across many host species, as is the case with nematode worms in *Myrmica* ants [12,18]. Finally, (iv) *Temnothorax* colonies are monogynous, so colony members are mostly full sisters. Hence, a high level of relatedness, which is key to minimizing genetic diversity’s effect in the detected morphological differences, can be assumed within the colony. Thereby, infection remains the most likely explanatory variable that has caused the morphological shifts.

## 2. Materials and Methods

### 2.1. Sampling

**Figure 1:**
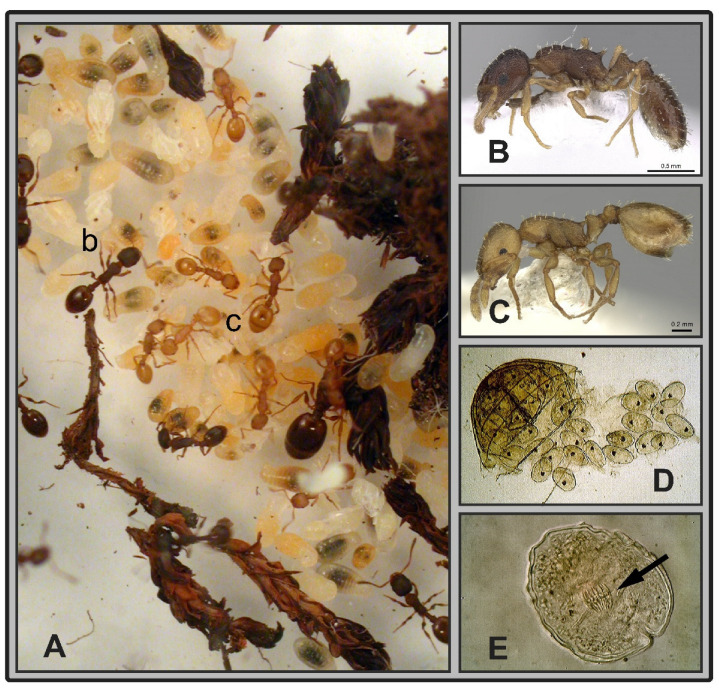
(**A**) Zoom on a colony of *Temnothorax sordidulus* with darker healthy workers (b) and lighter-colored infected workers (c) standing on the brood next to the queen. (**B**) and (**C**) Details of these healthy and infected workers. (**D**) Several parasite larvae in the gaster of an infected worker. (**E**) Morphological details of a parasite larva under high magnification with its hook wreath typical of tapeworm larvae (arrow).

We collected five parasitized colonies of three *Temnothorax* ant species in France (*T. nylanderi*: 3 colonies, Fontainebleau, 48.434056, 2.731333, alt 108 m), Croatia (*T. sordidulus*: 1 colony, Tucepi, 43.2744, 17.0539, alt 40 m), and Hungary (*T. unifasciatus*: 1 colony, Tatabánya, 47.5836, 18.4216, alt 254 m) and we stored them in 95% ethanol. Each sample was shipped to Hungary where the further processing, determinations, and dissections were made. Each individual was dissected to confirm their infection status (see below). All colonies contained both infected and uninfected individuals (Figure 1). Eighty-one individuals were used for morphometry: 39 *T. nylanderi* individuals (19 infected and 20 uninfected), 25 *T. sordidulus* individuals (10 infected and 15 uninfected), and 17 *T. unifasciatus* individuals (6 infected and 11 uninfected). Examined specimens are deposited in the Hymenoptera collection of the Hungarian Natural History Museum and the private collection of Sándor Csősz, Eötvös Loránd University, Budapest.

### 2.2. Identification of Infected Specimens

All individuals were dissected. We only found parasites in their abdomen, as white spheres with a diameter of 100–160 μm (Figure 1D). The parasites were white spheres with a diameter of 100–160 μm in the insects fixed in alcohol, and they deformed to a slightly oval shape due to the pressure of the coverslip placed on the microscope slide. After detachment of the posterior half of the abdomen, they could be easily removed from the host’s body, so they did not grow close to the body wall. However, thin pieces of tissue or pieces of tissue were attached to them, so it can be assumed that they developed in the body cavity but were attached to the intestinal wall or another organ. The parasites collected from ants were fixed in 70% ethanol solution, then placed in an aqueous solution containing 10% lactic acid and 50% glycerol in a shallow bowl where they remained for one or two days until they became translucent. Finally, the translucent parasites were mounted in 98% glycerol on microscope slides and covered with a coverslip (Figure 1E). In the middle of them, somewhat eccentrically, a hook wreath with a lateral protrusion characteristic of the rostellum of tapeworm larvae was observed via Alpha XJL-2R microscope. Rostellum is used by parasites to attach to the inside of the intestine of their host. The tapeworm larvae were identified as metacestode by the presence of ten, regularly 20 to 24 µm long hooks and four suckers. Suckers were observed mainly at the base of the rostellum squeezed from the parasite. These tapeworm larvae (also called cysticercoids), most likely belong to the order Cyclophyllidea of the subclass Eucestoda based on their location and internal structure. The larvae’s small hook wreath and hookless suckers suggest that they belong to the Hymenolepididae family. This family includes hundreds of tapeworm species, for which birds are usually definitive hosts, and arthropods are intermediate hosts.

### 2.3. Morphometrics

All trait measurements were made with an ocular micrometer installed on an Olympus SZX16 stereomicroscope at a magnification of 150x by F.B. All measurements were made in μm using a pin-holding stage, permitting rotations around X, Y, and Z axes. Morphometric data are given in μm and provided in Supplementary File S1 and is also available at https://figshare.com/articles/dataset/Temnothorax_morph_data/19596454/1, accessed on 14 April 2022.

Measured traits are defined as in [24] and abbreviated as follows:

CL: maximum cephalic length in median line; the head must be carefully tilted to the position with the true maximum. Excavations of hind vertex and/or clypeus reduce CL.

CWb: maximum width of head capsule, measured just posterior to the eyes.

EL: maximum diameter of the eye.

FRS: distance of the frontal carinae immediately caudal of the posterior intersection points between frontal carinae and the lamellae dorsal to the torulus. If these dorsal lamellae do not laterally surpass the frontal carinae, the deepest point of scape corner pits may be taken as a reference line. These pits take up the inner corner of the scape base when the scape is entirely switched caudad and produce a dark triangular shadow in the lateral frontal lobes immediately posterior to the dorsal lamellae of the scape joint capsule.

ML: mesosoma length from the caudalmost point of the propodeal lobe to transition point between anterior pronotal slope and anterior propodeal shield (preferentially measured in lateral view; if the transition point is not well defined, use dorsal view and take the center of the dark-shaded borderline between pronotal slope and pronotal shield as the anterior reference point).

MW: maximum mesosoma width.

NOH: maximum height of the petiolar node, measured in lateral view from the uppermost point of the petiolar node perpendicular to a reference line set from the petiolar spiracle to the imaginary midpoint of the transition between dorsocaudal slope and dorsal profile of caudal cylinder of the petiole.

NOL: length of the petiolar node, measured in lateral view from petiolar spiracle to the dorsocaudal corner of the caudal cylinder. Do not erroneously take as a reference point the dorsocaudal corner of the helcium, which is sometimes visible.

PEH: maximum petiole height. The chord of the ventral petiolar profile at node level is the reference line perpendicular to which the maximum height of the petiole is measured.

PEW: maximum width of the petiole.

PoOC: postocular distance. Use a cross-scaled ocular micrometer and adjust the head to the measuring position of CL. Caudal measuring point: median occipital margin; frontal measuring point: median head at the level of the posterior eye margin.

PPH: maximum height of the postpetiole in lateral view measured perpendicularly to a line defined by the linear section of the segment border between dorsal and ventral petiolar sclerite.

PPL: maximum length of the postpetiole measured in a lateral view perpendicular to the straight section of lateral postpetiole margin.

PPW: maximum width of postpetiole.

SL: maximum straight-line scape length excluding the articular condyle.

SPL: minimum distance between the center of propodeal spiracle and the subspinal excavation measured in lateral view (i.e., the same view applied to measure ML). Note: in lateral view, propodeal spiracle and the caudal margin of propodeal declivity might not be in the same focal level; hence, slight adjust might be necessary while measuring SPL between the two endpoints.

SPBA: the smallest distance of the lateral margins of the spines at their base. This should be measured in the dorsofrontal view since the broader parts of the ventral propodeum do not interfere with the measurement in this position. If the lateral margins of spines diverge continuously from the tip to the base, the smallest distance at the base is not defined. In this case, SPBA is measured at the level of the bottom of the interspinal meniscus.

SPST: distance between the center of propodeal stigma and spine tip. The stigma center refers to the midpoint defined by the outer cuticular ring but not to the center of the real stigma opening that may be positioned eccentrically.

SPTI: the distance of spine tips in dorsal view; if spine tips are rounded or truncated, the centers of spine tips are taken as reference points.

SPWI: maximum distance between outer margins of spines; measured in the same position as SPBA.

PEL: diagonal petiolar length in lateral view; measured from anterior corner of the subpetiolar process to the dorsocaudal corner of the caudal cylinder.

## 3. Data Analyses

Data handling and statistical analyses were performed using R software (ver. 4.0.1) [25]. We used principal component analysis (PCA) to reduce the number of variables representing morphological variation, retaining the first two axes for visualization. In a preliminary analysis step to test if infection affected morphological traits and whether the direction and magnitude were similar among species and traits, we fitted a linear regression model with measurements of morphological traits as the response variable, and infection status, species, and names of traits (as defined in the Morphometrics section) as predictors, with control to their 2- and 3-way interactions. From the analysis of variance tables, we identified whether or not interaction terms were significant, which would indicate that (1) infection affects different morphological traits differently, and/or (2) the effect of infection differs between species. Subsequently, we fitted linear regression models separately for each measured morphological trait (i.e., individual models for each trait) to get marginal estimates for trait values per species and infection status. The measurements of each trait are the response variable, and the categorical predictors are species (three levels: *T. nylanderi*, *T. sordidulus*, *T. unifasciatus*), infection status (two levels infected, uninfected), and their interaction. Prior to model fitting, we re-scaled morphological trait values by subtracting the arithmetic mean from all values then dividing by the standard deviation separately for each species to exclude the confounding effect of species-specific body sizes in the models’ parameter estimates. Following model fitting, we again used analysis of variance tables on the models to acquire estimates for the interaction term between species and infection was significant. *p*-values for the interaction terms were adjusted with Bonferroni’s method to decrease the probability of type I errors occurring. Also, we extracted contrast parameters between infected and uninfected specimens using the “emmeans” R-package [26]; *p*-values for the estimated contrast parameters were adjusted with Bonferroni’s method. Finally, we visually checked for homoscedasticity and normal distribution of the residuals for all linear models using plot functions.

We used the morphology and infection data to train an artificial neural network-based multiclass classifier model, which could predict species merely based on morphological trait values and the presence of parasite infection. Machine learning-based approaches are already used for image-based species identification [27], and utilizing trait data in multiclass classification problems has been successful in ants [28]. Admittedly, our approach is an intermediate to these approaches since we used phenotypic data directly (rather than from images) for species identification, while still relying on a machine learning approach [29] as the high-dimensional and structured nature of complex phenotypic, data is substantially more challenging to model using classical statistical methods. The model matrix was built so that predictors were the morphological traits, infection status, and the interactions between each trait and infection. The topology of the neural network (NN) consisted of three hidden layers, each with ten hidden units. Model fitting was done using backpropagation via gradient descent, utilizing logistic activation functions on the NN nodes, and cross-entropy loss function. (The codes for the used R implementation are available at github: https://github.com/zradai/R/tree/master/MachineLearning, accessed on 14 April 2022). We applied a 10-fold cross-validation to assess how well the fitted models can generalize to data that they were not acquainted with. In the cross-validation iterations, we used stratified random re-sampling to train the models to keep the same relative proportions of species in the training data sets across model fitting. Finally, to quantify model performances, we calculated the classical performance measures of classification algorithms (namely: accuracy, precision, sensitivity, specificity), as well as the Matthews correlation coefficient, from the predictions of the models on the test data (i.e., on data which were not involved in model training).

Subsequently, we estimated the putative uninfected trait values for infected specimens by considering the species-specific distributions of trait values, separately for infected and uninfected ants. First, we estimated mean and standard deviation of trait value distributions with maximum likelihood, separately for infected (*μ_inf_*, *σ_inf_*) and uninfected (*μ_un_*, *σ_un_*) phenotypes within species. Then, we calculated the standardized distance from the mean infected trait value for each infected specimen as *d = (y − μ_inf_)/σ_inf_*, with *y* the given infected specimen’s observed trait value. Afterward, we calculated the predicted wild-type trait value as *p = d × σ_un_ + μ_inf_ + s*, with *s* the contrast parameter representing the difference between wild-type and infected mean trait values, i.e., *s = (μ_un_ − μ_inf_)*. The script is written in R and is available in Supplementary File S2 and at https://github.com/zradai/R/blob/master/published_research_analyses/Morphological_reconstruction_2022/R_analyses_Csosz_et_al_2022.R, accessed on 14 April 2022.

## 4. Results

**Figure 2:**
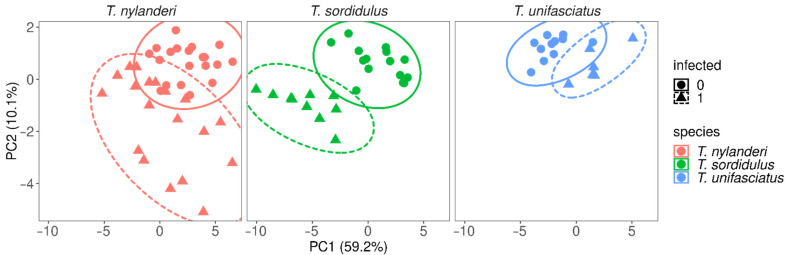
Principal component analyses representing the assessed morphological traits, separately for *T. nylanderi* (**left**), *T. sordidulus* (**middle**), and *T. unifasciatus* (**right**); uninfected and infected individuals are indicated by circular and triangular shapes, respectively.

**Figure 3:**
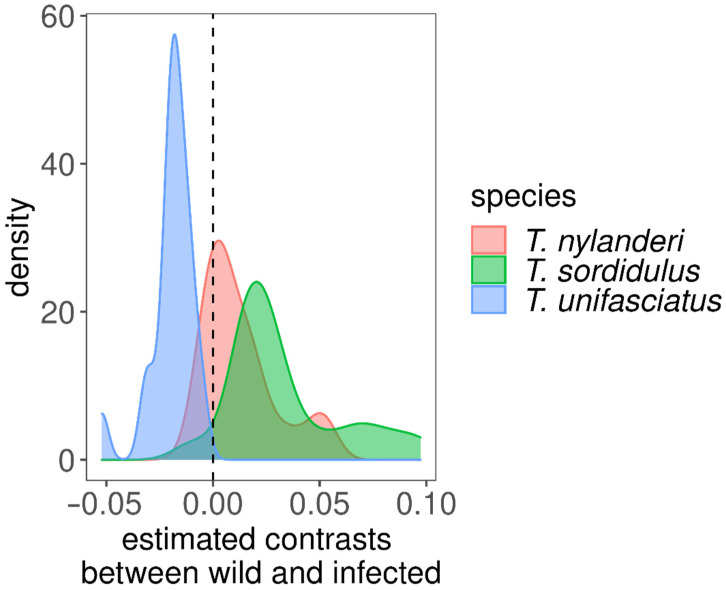
Density-kernel plot of estimated contrast parameters from the comparisons of uninfected and infected ants, separately for species. In *T. nylanderi* and *T. sordidulus*, infected specimens had smaller trait values; whereas, in *T. unifasciatus*, infected specimens had smaller trait values.

Based on our first fitted model, trait values were affected differently by infestation across traits and species, as indicated by the significant interaction terms between trait, infection status, and species (Table 1). Indeed, when the effect of infection was modeled separately for traits, the studied species showed substantially different morphological responses to infection, as we found significant interaction terms between species and infection for all morphological traits (Table 1, Figure 2). However, in the case of one trait (SPBA), the *p*-value rose above 0.05 (to *p* = 0.256) after Bonferroni’s adjustment (Supplementary Table S3). Traits were significantly smaller in infected than in uninfected individuals in *T. nylanderi* and *T. sordidulus*, but we found the opposite in *T. unifasciatus* (Supplementary Table S3, Figure 3).

**Figure 4:**
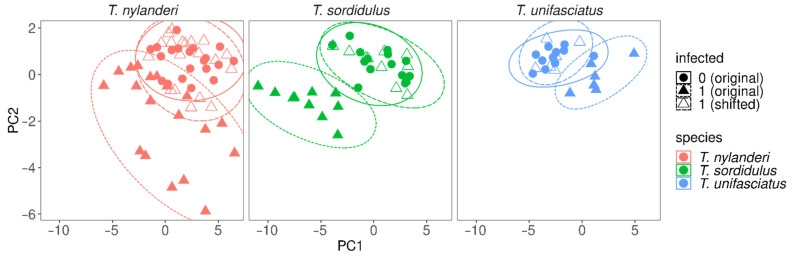
Principal component analyses representing the assessed morphological traits, separately for *T. nylanderi* (**left**), *T. sordidulus* (**middle**), and *T. unifasciatus* (**right**); uninfected and infected individuals are indicated by circular and triangular shapes, respectively; empty triangles represent predicted uninfected trait values estimated from infected individuals, i.e., trait values that originated from infected specimens, but were transformed based on the modelled trait-differences between uninfected and infected phenotypes.

The 10-fold cross-validation showed excellent prediction power of fitted NN classifiers, as shown by the performance measures (Table 2), meaning that the classifier could identify species based on morphology and presence of infection with very high certainty. Furthermore, the predicted wild-type trait values estimated based on the trait value distributions showed that reconstruction of trait values of infected specimens yields values closely resembling those of wild-type ants (Figure 4).

## 5. Discussion

Our morphometric analysis has shown that the cysticercoid infection causes severe and predictable morphological changes in host individuals of each investigated *Temnothorax* ant species. The 10-fold cross-validation showed excellent prediction power of fitted NN classifiers, as shown by the performance measures, meaning that the classifier could identify species based on morphology and presence of infection with very high certainty. The predicted uninfected trait values estimated based on the trait value distributions showed that reconstruction of trait values of infected specimens yields values closely resembling those of uninfected ants. Thereby, infected individuals can be adequately transformed into their uninfected equivalent of the same species. These parasitogenic phenotypes differ from the conspecific uninfected individuals in overall body size and shape, and these changes are species-specific. While the infection caused a decrease in the size of most measured traits in *T. sordidulus* and *T. nylanderi*, the infected individuals were larger in the case of *T. unifasciatus*. This observation is also remarkable because, in the past, most research has focused on one host species only, *T. nylanderi* [13,30], so our study shows that the parasite may have a different effect even in closely related species. Therefore, species-specific data about how parasite infection affects morphological traits is required to identify the species associated with an unknown infected individual. Our method can thus only be applied to species from which morphological measurements of wild-type and infected specimens are available. This limitation found in the studied *Temnothorax*-Cestode system might be applied to host-parasites systems with other ant genera (as host organisms) and even outside ants. A more extensive taxonomic survey is, thus, required. Unfortunately, the reasons for this phenomenon can be multifaceted. They might be ascribed to a great variety of factors, including different genetic pools, differences in developmental dynamics, various environmental factors, different lifestyles of host species, the diversity of colony-level traits (e.g., number of individuals, age of the colony), or to the taxonomic diversity of the parasites.

Different parasite species may cause different changes. In our research, we could not identify the parasite larva at the species level. However, the small hook wreath of the larvae and hookless suckers suggest that these are most likely members of the Hymenolepididae family. This family includes hundreds of tapeworm species, mainly in birds, and the species of this family usually develop in arthropods as larvae [31,32,33]. Larvae cannot be more precisely identified based on anatomical structures, but previous studies suggest that European *Temnothorax* species are infected by *Anomoteania brevis* (Clerc 1902) [34,35,36]. However, we cannot be sure that the parasites of the three ant species we studied belong to the same species. The intensity of infection, i.e., the number of parasite larvae and the time scale of infection that might affect larval growth, were also out of the scope of this study.

Laboratory research would be required to infect colonies with a single strain of parasites, and diverse environmental factors must be standardized to eliminate the effects of these variables. However, many of these parasites have complex life histories, comprising two phases: a stage in a definitive (primary) host (which is a vertebrate) in which the adults develop and reproduce, often for years, and an intermediate stage in which the larvae grow in other hosts, in our case, ants. The complex life cycle of the Cestode parasites makes running long-term laboratory experiments extremely challenging. Moreover, because the parasite larvae cannot be routinely determined easily down to the species, and because the life stage of the ant colonies at the time of collection is usually unknown, its benefit in routinely applied taxonomic workflow would be limited. Due to the above reasons, laboratory research was out of the scope of our current study. However, we believe that such a long-term laboratory experiment would illuminate unexpected new phenomena. Furthermore, such parasite-mediated developmental perturbations would provide exciting tests of recent models for the developmental mechanisms generating trait allometries in insects [37,38]. In addition, the adaptive nature of the induced morphological shifts for the parasite remains to be explored using evolutionary ecology, developmental biology, and eco-physiology approaches.

Our work sheds light on the integrability of alternative parasitogenic phenotypes in morphometric analyses at the species level. Modeling hosts’ morphological shifts caused by parasites can be a powerful method for taxonomists, especially in species where samples are scarce or heavily parasitized. Beyond the currently presented application of our concept, i.e., transforming parasitogenic morphologies into a virtual, healthy one, our approach might be used in systems where polyphenism is high. Thereby, this method can also be used in integrating alternative phenotypes in ant genera where workers are divided into two distinct subcastes, minor and major workers, called soldiers (e.g., *Eciton*, *Pheidole*), dimorphic [39], or trimorphic beetles [40], or other insects where a high level of polyphenism leads to clusters of conspecific individuals with a highly different appearance.

## Figures and Tables

**Table 1:** ANOVA table from the linear regression model on trait measurement, using trait, infestation, and species as predictors (see Data Analyses), shows the variation components per the used predictors and their interaction terms.

Component	Df	Sum Sq	Mean Sq	F Value	Pr (>F)
trait	20	48.798	2.440	8921.731	0.000
as. factor (infected)	1	0.056	0.056	205.174	0.000
species	2	0.076	0.038	139.383	0.000
trait: as. factor (infected)	20	0.086	0.004	15.711	0.000
trait: species	40	0.117	0.003	10.707	0.000
as. factor (infected): species	2	0.131	0.066	239.836	0.000
trait: as. factor (infected): species	40	0.059	0.001	5.394	0.000
Residuals	1575	0.431	0.000	NA	NA

**Table 2:** Performance measures of the neural network multiclass classifier, from the 10-fold cross-validation (MCC stands for Mathews correlation coefficient).

kth Model	Accuracy	Precision	Sensitivity	Specificity	MCC
1	1.00	1.00	1.00	1.00	1.00
2	1.00	1.00	1.00	1.00	1.00
3	1.00	1.00	1.00	1.00	1.00
4	1.00	1.00	1.00	1.00	1.00
5	0.98	0.95	1.00	0.98	0.97
6	1.00	1.00	1.00	1.00	1.00
7	1.00	1.00	1.00	1.00	1.00
8	0.98	0.95	1.00	0.98	0.97
9	0.97	0.95	0.95	0.98	0.93
10	1.00	1.00	1.00	1.00	1.00
averaged	0.99	0.99	1.00	0.99	0.99

## Supplementary Materials

The following supporting information can be downloaded at: https://www.mdpi.com/article/10.3390/life12050625/s1, Table S1. Raw morphometric data for individuals. Table S2. R-script for predicting wild-type presumptive trait values for infected individuals. Table S3. ANOVA table from the linear regression model on trait measurement, using trait, infestation, and species as predictors. The effect of infection was modeled separately for traits.
